# Distance Decay of Similarity in Neotropical Diatom Communities

**DOI:** 10.1371/journal.pone.0045071

**Published:** 2012-09-13

**Authors:** Carlos E. Wetzel, Denise de C. Bicudo, Luc Ector, Eduardo A. Lobo, Janne Soininen, Victor L. Landeiro, Luis M. Bini

**Affiliations:** 1 Núcleo de Ecologia, Instituto de Botânica, São Paulo, SP, Brazil; 2 Department of Environment and Agro-Biotechnologies, Public Research Centre - Gabriel Lippmann, Belvaux, Luxembourg; 3 Laboratory of Limnology, Universidade de Santa Cruz do Sul, Santa Cruz do Sul, RS, Brazil; 4 Department of Geosciences and Geography, University of Helsinki, Finland; 5 Departamento de Ecologia,Instituto de Ciências Biológicas, Universidade Federal de Goiás, Goiânia, GO, Brazil; National Institute of Water & Atmospheric Research, New Zealand

## Abstract

**Background:**

The regression of similarity against distance unites several ecological phenomena, and thus provides a highly useful approach for illustrating the spatial turnover across sites. Our aim was to test whether the rates of decay in community similarity differ between diatom growth forms suggested to show different dispersal ability. We hypothesized that the diatom group with lower dispersal ability (i.e. periphyton) would show higher distance decay rates than a group with higher dispersal ability (i.e. plankton).

**Methods/Principal findings:**

Periphyton and phytoplankton samples were gathered at sites distributed over an area of approximately 800 km length in the Negro River, Amazon basin, Brazil, South America (3°08′00″S; 59°54′30″W). Distance decay relationships were then estimated using distance-based regressions, and the coefficients of these regressions were compared among the groups with different dispersal abilities to assess our predictions. We found evidence that different tributaries and reaches of the Negro River harbor different diatom communities. As expected, the rates of distance decay in community similarity were higher for periphyton than for phytoplankton indicating the lower dispersal ability of periphytic taxa.

**Conclusions/Significance:**

Our study demonstrates that the comparison of distance decay relationships among taxa with similar ecological requirements, but with different growth form and thus dispersal ability provides a sound approach to evaluate the effects of dispersal ability on beta diversity patterns. Our results are also in line with the growing body of evidence indicating that microorganisms exhibit biogeographic patterns. Finally, we underscore that clumbing all microbial taxa into one group may be a flawed approach to test whether microbes exhibit biogeographic patterns.

## Introduction

In a recent review it was suggested that diversity could be partitioned into two different components. The first component, inventory diversity, relates to the species composition of a single plot or a region and thus refers to classical Whittake?s alpha and gamma diversity; the second component considers changes in species composition between plots or areas and is known as beta diversity or differentiation diversity [1]. In recent years, different methods to analyze patterns in beta diversity have been developed, given the paramount importance of this component in both theoretical and applied terms [2]–[4]. In 1970, W. Tobler stated what is regarded as the first law of geography: “Everything is related to everything else, but near things are more related than distant things [5].” Inspired by Tobler’s work, botanists popularized a pattern that is currently recognized as the distance decay of similarity (DDS) [6]. This pattern emerges when compositional similarities (i.e., the complement of beta diversity) decrease with the increase of geographic distances between sites. Currently, the DDS is one of the most widely studied relationships in ecology and has been recognized as a manifestation of processes controlling community composition on different spatial scales [7]–[8].

Nekola & Whitte [6] provided a comprehensive conceptual framework of distance decay rate variation. According to them, the DDS can be explained by different extrinsic and intrinsic factors. Extrinsic factors include, for instance, environmental variability, habitat isolation and dispersal barriers, whereas intrinsic factors include dispersal ability, body size, trophic position and thermoregulation of the organisms concerned [7], [9]. Dispersal ability, in particular, is a key factor that could explain much of the variation in the rate of the DDS. For instance, a high rate of similarity decay can be expected for organisms with low dispersal ability [6], [10]–[11].

Many organism groups offer interesting possibilities to study the potential effect of dispersal ability on patterns in DDS. Diatoms (Bacillariophyceae) belong to a diverse group of microalgae. Depending on the species concept, conservative estimates suggest that the total number of diatom species could be as high as two hundred thousand [12]. They have a worldwide distribution and constitute a major component of planktonic and benthic algal communities from oceanic waters to polar ice caps and from moist soils to freshwater alkaline lakes or eutrophic estuaries [13]. They form a taxonomically dominant and functionally diverse community in streams and rivers, possessing different growth forms and strategies to resist environmental pressures, such as grazing and flow disturbance. The different life forms include benthic, planktonic, mobile, colonial, mucous tubule, pedunculate and pioneer taxa [14]. All these strategies are supposedly connected with their dispersal abilities and, consequently, with the patterns in beta diversity exhibited by these organisms.

Some authors have emphasized that, in general, diatoms are ubiquitous and their community compositions are predominantly determined by species sorting of the environment [15]–[16]. According to Finlay et al. [17], diatoms possess exceptional dispersal abilities because many taxa have a cosmopolitan distribution, and undersampling of rare habitats, taxa or resting stages have led to erroneous claims of endemism. However, several fine-grained taxonomic studies suggest endemism, geographic limits and dispersal constraints for diatoms [18]–[20]. Indeed, there is accumulating evidence that diatoms also respond to large-scale climatic, historical or dispersal-related factors [21]–[22].

In this study, we examined the DDS in periphytic and planktonic diatom communities from one of the largest neotropical rivers (the Negro River, a Brazilian tributary of the Amazon) to study whether the rates of decay in similarity differ between the diatom groups growing in different habitats. Building on the conceptual framework developed by Nekola & White [6], we expected a faster decrease of similarity with distance for periphytic species (which possess poorer dispersal abilities due to their association with the substrata) than for free-living planktonic species (which possess higher dispersal abilities). Following a deconstructive approach [23], we also tested the same prediction after dividing each of the communities according to inferred dispersal abilities.

## Materials and Methods

### Study Area

**Figure 1:**
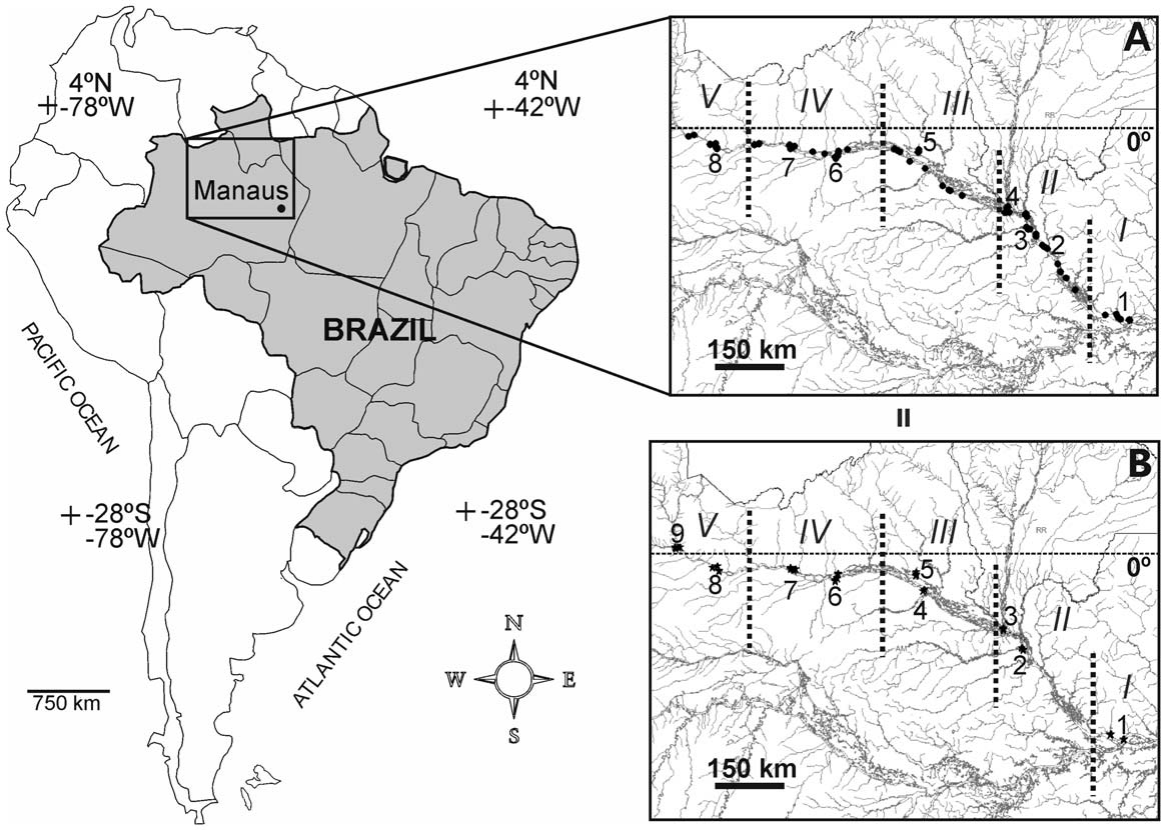
Sampling sites located along the Negro River hydrographical basin, the Brazilian Amazon for (A) phytoplankton samples (*n* = 114), including tributaries (1–8) and the main channel; and for (B) periphyton samples collected on tributaries (1–9) (*n = *129). Geomorphological reaches (I to V) are presented according to Latrubesse and Franzinelli [24].

Of the hundreds of tributaries of the Amazon River (15°31′5″S; 71°45′55″W), the Negro River is considered to be one of the most important, due to its high flow (annual mean ≈ 29,000 m^3^ s^−1^), being the second largest discharge tributary [24]. Its basin mostly encompasses an intensely weathered area of the Precambrian shield and extends 1,100 km in the east-west direction and 600 km in the north-south direction with a basin area of 715,000 km^2^. The hydrographical basin of the Negro River is characterized by environments with low nutrient concentrations and pH values [25]. Exceptions, such as the Branco River, have intermediate values between black and white waters for most limnological variables (i.e., pH, conductivity, dissolved organic carbon, metals and ionic composition). The Branco River is a ‘clear’ water river and is one of the most important affluents on the left bank of the Negro River in its middle-low reach. According to geomorphological characteristics, the Negro River basin can be divided into six reaches [24], five of which were sampled during our study (Figure 1).

### Sampling

During an expedition across the Negro River basin in March 2005, two different sets of samples (periphyton and phytoplankton) were collected. Samples were gathered during the rising water period. Subsurface phytoplankton samples were collected from the main channel of the Negro River and its tributaries (*n = *114, Figure 1, A) with a plankton net (54 µm mesh) and were fixed with a solution of 3–4% formaldehyde. Periphytic samples (*n = *129) were collected mainly in riparian flooded forests located in nine different tributaries (Figure 1, B). Samples were gathered mainly from litter and submerged plants (*n = *119). A small number of samples (*n = *10) were collected from other microenvironments (rocks and sand sediments). All necessary permits were obtained for the described field studies. They were not conducted in privately-owned or protected area and did not involved endangered or protected species.

### Sample Treatment

Homogenized aliquots of the material from both periphyton and phytoplankton samples were heated in an 85 °C water bath with a 30% hydrogen peroxide (H_2_O_2_) solution for at least 3 hours to oxidize the organic matter and clean the frustules. Next, samples were cleaned with 10% hydrochloric acid (HCl) to dissolve carbonates. Cleaned samples were mounted on glass slides using Naphrax® as mounting medium. Slides were counted on a Zeiss® (Axioscope® 2) at 1000X magnification under oil immersion. Relative abundances were estimated following the counting technique described by Pappas and Stoermer [26] with a minimum of 400 valves counted per slide on up to six random transects until reaching an efficiency ≥92%.

### Species Traits used to Deconstruct Phytoplankton and Benthic Communities

The species matrix was divided into four main tables based on different studies (Text S1). Thus, we further split the phytoplankton species matrix into true planktonic (TP) and loosely attached mobile species (MP). Similarly, the benthic species data table was divided into loosely attached mobile taxa (MB) and firmly attached species (true benthic TB). Within each community (i.e., planktonic and benthic), we assumed that vagility was higher for TP and MB when compared with MP and TB, respectively. According to our initial hypothesis, the rate of decline in similarity with distance should be greater for MP than for TP. A similar pattern should be found for the comparison between TB and MB. As these groups possess similar environmental requirements, we assumed that this scheme of comparison could, at least partially, rule out the possibility that differences in the patterns of distance decay could be solely or mostly attributed to differences in the ways that the groups respond to species sorting mechanisms; the differences could then be better explained by dispersal abilities. We thus assumed higher dispersal ability for phytoplankton community than for periphytic diatoms, which adhere to substrata. Within periphyton and phytoplankton, dispersal ability was assumed to increase from TB to MB and from MP to TP.

### Turnover and Nestedness

Patterns in beta diversity can be decoupled into a spatial turnover component (variation in species identity among sites) and a nestedness component (when sites with lower richness are subsets of sites with higher richness; see [27]. To quantify the relative importance of these components, we used the procedures described by Baselga [28].

### Initial Similarity and Distance Decay Analyses

For each matrix (periphyton, phytoplankton and deconstructed sets), we used the Bray-Curtis index of similarity to calculate pairwise similarities of abundance data between sampling sites. A matrix of geographic (Euclidean) distances between sites was derived from the longitudinal and latitudinal coordinates. We used euclidean distances instead of distances along river channels because diatoms disperse efficiently also via air. Subsequently and for each algal group, the Bray-Curtis distance matrix was regressed on the geographic distance matrix [29]. We used the intercept of the resulting regression model as a measure of the “initial similarity” [6]–[7]. A high intercept indicates a low level of beta diversity at short distances (as expected for organisms with high vagility). The standardized regression coefficients of these regression models were used as measures of the rate of decay in similarity as a function of geographical distance between sites. As we were using only one explanatory variable, the standardized regression coefficients we obtained were identical to the Mantel's matrix correlation statistics. *P*-values associated with the regression coefficients were estimated using 10,000 randomizations [29]. To test whether the rates of decay in similarity differ between the diatom groups we used a Monte Carlo randomization procedure based on Nekola & White [6]. The procedure consisted of the following steps: (i) the lower triangles of the similarity matrices (one for each group under comparison) were unfolded into a single vector containing (*n*_1_×*n*_1_–1)/2+ (*n*_2_×*n*_2_–1)/2 rows (where *n*_1_ *and n*_2_ are the number of sites for the groups under comparison); (ii) the slope of the relationship between geographic distance and community similarity was estimated for each group; (iii) a vector with the same number of rows and containing the codes to identify the groups under comparison was created (e.g., TB and MB) and paired with the similarity vector; (iv) the vector containing the labels was randomly rearranged 10000 times and, for each randomization, the absolute difference between the slopes were calculated (the criterion statistics) to produce a null distribution for the absolute differences between the slopes; (v) the *P*-values were estimated as the ratio between the number of times that the randomized differences were equal or greater than the observed difference (+1) and the number of randomizations. The number of zero similarity values was very low (eight for periphyton and 95 for phytoplankton) and had no effect on the analysis; therefore, we included zero similarity values in all analyses. All distance decay analyses were repeated after transforming abundance data into presence/absence data. In these cases, we used the pairwise Simpson index to calculate the floristic similarities between sampling sites [28] (Figure S1). For comparative purposes, we also estimated initial similarity values following the procedures described by Soininen [7]. All statistical analyses were performed in R (R Development Core Team [30]) using the libraries *vegan* and *ecodist* and the functions provided by Baselga [28]. The Monte Carlo randomization procedure was written in R language and is available upon request.

## Results

### Diatom Composition and Diversity

A high number of diatom taxa (652) was identified in the samples (*n* = 243 samples). Eunotiaceae was the most species-rich family with almost a third (28.5%) of the species from both periphytic and planktonic environments [31] followed by Pinnulariaceae (14.6%), Naviculaceae (8.6%), Cymbellaceae (8.0%), Sellaphoraceae (7.1%), Gomphonemataceae (5.0%), Surirellaceae (4.8%) and Fragilariaceae (3.2%). These families represented 79.2% of the total species number detected. *Aulacoseira* Thwaites, *Asterionella* Hassal, *Fragilariforma* D.M. Williams et Round, small-celled (±10 µm) genera (e.g., *Chamaepinnularia* Lange-Bertalot et Krammer, *Eolimna* Lange-Bertalot et W. Schiller, *Nupela* Vyverman et Compère) and the family Stephanodiscaceae were among the most abundant taxa in phytoplankton samples. Periphyton communities were mainly represented by the members of Eunotiaceae (*Eunotia* Ehrenberg with 151 species and *Actinella* F.W. Lewis with 31 species) (Table S1 and Figure S2).

### Nestedness and Community Turnover

**Figure 2:**
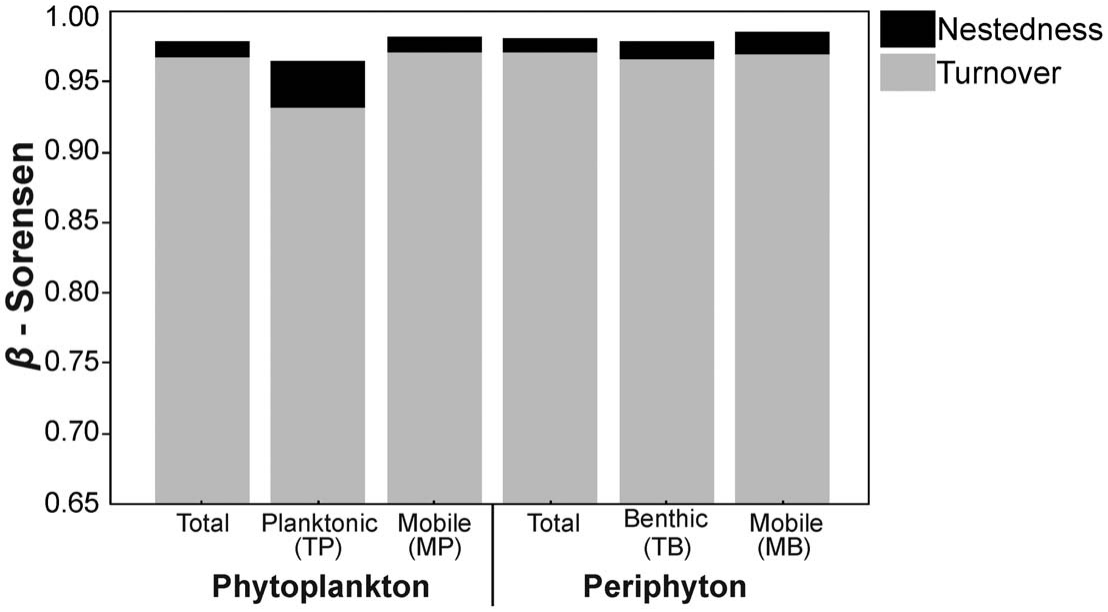
Turnover and nestedness components of beta diversity for the different diatom groups.

Patterns of beta diversity in phytoplankton and periphyton at the Negro River hydrographical basin were mainly caused by the spatial turnover with a small contribution from nestedness (Figure 2). A high nestedness component of beta diversity was observed only for true planktonic species (β_NES_ = 0.034), but this component was still much lower than the turnover component (β_SIM_ = 0.930).

### Distance Decay Relationships (DDR)

**Figure 3:**
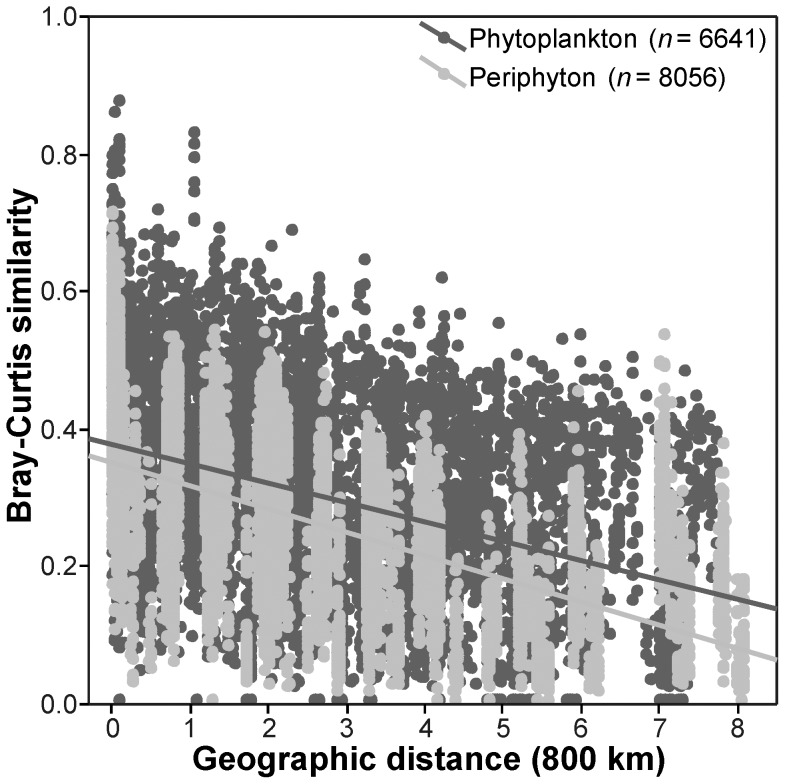
Bray-Curtis similarity of phytoplankton and periphyton samples plotted against geographical distance between sites.

**Figure 4:**
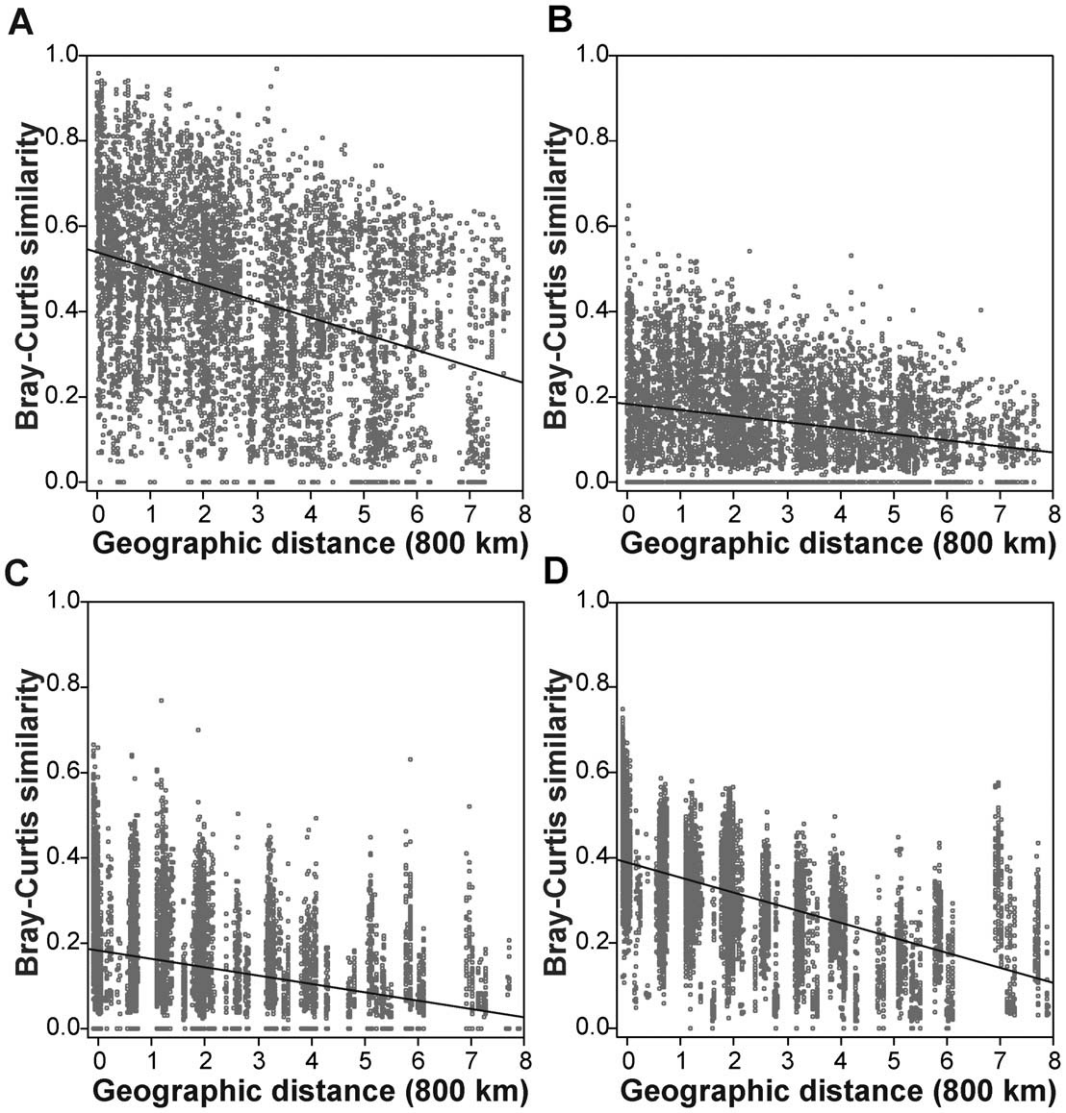
Bray-Curtis similarity of phytoplanktonic (A-B) and periphytic diatom communities (C-D) plotted against geographical distances between sites. A. True planktonic species (TP); **B.** mobile planktonic species (MP); **C.** Loosely attached mobile taxa (MB); **D.** Firmly attached species (true benthic TB).

In all data sets, community similarity decreased significantly with distance (*P*<0.001, Figure 3 and 4). However, there were important differences in the distance decay relationships (DDS) among the groups under comparison, as described by their intercepts, slopes and levels of scatter. For instance, the DDS for all species recorded in phytoplankton samples showed a significantly (*P*<0.001) lower slope (standardized regression coefficient *b* = −0.38±0.01 SE), a higher intercept (“initial similarity” *a* = 0.38±0.003) and a lower coefficient of determination (*R* ^2^ = 0.15) when compared to the DDS obtained for periphyton communities (*b* = −0.56±0.009; *a* = 0.35±0.002; *R* ^2^ = 0.31; Figure 3). The DDS obtained for true planktonic diatoms (TP, Figure 4, A) showed a significantly (*P*<0.001) higher value of *a* (0.54±0.004) and *b* (−0.35±0.012) than the DDS derived from mobile species (MP; *a* = 0.18±0.002; *b* = −0.24±0.012; Figure 4, B). Considering the periphyton samples, the DDS for benthic diatoms (TB, Figure 4, D) also presented a significantly (*P*<0.001) higher *b* (−0.54±0.009) and *a* (0.39±0.002) when compared with the mobile group (MB; *b* = −0.30±0.011; *a* = 0.18±0.002) (Figure 4, C). The randomization procedure also revealed a significant difference (*P*<0.001) when the comparison was made between between TB and TP, but not between MB and MP (*P* = 0.547). In general, most of these results were in line with our predictions (Figure S1), and similar results were found for the Simpson index applied to the incidence-based data (Figure S3).

## Discussion

Our basic results agree with what is expected from a megadiverse region. Besides several taxa that are known from other regions in similar habitats, we found several populations that either did not entirely fit the descriptions found in floras from Europe, North America or elsewhere, or that could not be related to any taxa known to us at the time of the preparation of this paper. While it is possible that some undetermined taxa could be linked to a published name after extensive literature searches and comparison with type material, others are potentially new species. Species accumulation curves (unpublished results) indicated that the actual species richness of this region is far greater than the 652 diatom taxa identified in our study. Although a few comparisons of species richness between regions cannot be definitive, it is nevertheless clear that the diversity of freshwater diatoms from the Neotropic is among the most diverse in the world.

### Diatom Beta Diversity Across the Negro River Watershed: How High was it in Relation to other Studies and How Predictable was it?

The magnitude of beta diversity estimated in a given study can be explained by the interaction of multiple intrinsic (e.g., dispersal ability of organisms) and extrinsic factors (e.g., latitudinal position, size and type of the system under study [6], [9]. Based on the results provided by the meta-analysis published by Soininen et al. [9], we can verify whether our results are within the expected range, considering the intrinsic and extrinsic factors they analyzed. More importantly, this comparison may shed some light on the relative importance of these factors in driving beta diversity patterns. Similarity values declined with increasing distance between sampling sites (as expected by Tobler’s first law of geography; see [32]. If compared with reported values in literature [33]–[34], these seem quite high indicating high species turnover in Neotropical communities. This suggests that large-scale turnover diversity may be high in low latitudes compared with regions nearer the poles [9].

For comparative purposes, interesting measure of beta diversity is also given by the “initial similarity” (the similarity at one km distance), and all results discussed in this section are based on this metric and on the Sørensen index [7]. The predicted initial similarities for phytoplankton and periphyton communities at the Negro River were 0.352 (CI_95%_ = 0.347–357) and 0.373 (CI_95%_ = 0.369–0.376), respectively. These low values were comparable to those reported by Soininen et al. [7] for microorganisms living in freshwater systems located at low latitudes (see their figures 2 h, 2b, and 2a) but lower than for planktonic communities in Finland [34]. High beta diversity (i.e., low initial similarity) for small organisms inhabiting freshwater systems localized at lower latitudes is expected if we assume, simultaneously, a predominance of small-bodied and range-restricted species near the tropics (which, due to higher surface-to-volume ratios and shorter generation time, are more sensitive to fine-scale environmental variability [35]) and that freshwater systems are more isolated than terrestrial systems. Taken together, these results indicate the importance of such factors as body size, realm and latitude in determining beta diversity values. More importantly, and independently of the underlying mechanisms, results from Soininen et al. [7] and our comparative analysis indicate that beta diversity patterns are, to some extent, predictable [36]. In addition, these results appear to be robust to the use of an alternative similarity index (see discussion between Baselga [27] and Soininen and Hillebrand [37]), as we also obtained a low initial similarity with the use of the Simpson index (CI_95%_ = 0.480–0.493 for phytoplankton and CI_95%_ = 0.478–0.487 for periphyton).

Considering our results, the comparison of the initial similarities (*y*-intercepts) estimated with the Simpson index, which provides estimates that are independent of species richness [27]–[28], [38], highlighted the importance of dispersal abilities in explaining beta diversity values. Indeed, we estimated the highest initial similarity (i.e., lowest small-scale beta diversity) for true planktonic species (*a* = 0.78; CI_95%_ = 0.769–789; see Figure S3). This result is in line with the expectation that high dispersal abilities (as assumed for true planktonic algae) tend to homogenize communities and to reduce beta diversity in small spatial scales [39]–[40].

### Do Diatoms have Biogeography?

Diatom beta diversity patterns across the Negro River watershed were mainly driven by spatial turnover. This result suggests that some level of biogeographic provincialism cannot be disregarded. Unfortunately, we do not have a comprehensive set of environmental variables because some basic limnological variables were measured only at certain sampling sites. In principle, we would need a more comprehensive dataset to fully verify whether the among-site differences in diatom composition were mostly due to biogeographic signals, rather than current species-sorting mechanisms. Although we are not ruling out the roles of species-sorting mechanisms by the unmeasured environmental variables, other lines of evidence suggest that dispersal limitation and barriers were also important in shaping the patterns we found. For example, most of the sampling sites were located in black water rivers (except those in the Branco River), which tend to be environmentally homogeneous in the Negro River hydrographical basin. Indeed, according to the data gathered simultaneously with the algal samples, the coefficients of variation estimated for pH, dissolved oxygen (*n* = 41) and ionic concentrations (as indicated by conductivity; *n* = 29) were relatively low (10.8%, 38.6% and 55.4%, respectively; see Table S2). Also, it is important to note that all samples were gathered during the high water period; even so, despite the fact that floods tend to homogenize the abiotic characteristics and the biota of aquatic habitats [41], we found that communities were spatially patterned.

There is an ever-increasing amount of evidence indicating that microbial organisms exhibit biogeographic patterns [42]–[44] (Green et al. 2004, Noguez et al. 2005, Telford et al. 2006). Thus, the studies previously cited, among others, support the second part of the L.G.M. Baas-Becking statement, “the environment selects,” but reject the first part that states “everything is everywhere” [44]–[46].

### What is the Role of Dispersal Ability on Distance Decay Patterns?

Nekola and White [6] were among the first to raise and test the hypothesis that increased dispersal ability would cause a decrease in distance decay rates. Specifically, using a dataset obtained in North American spruce-fir forests, they show, for instance, that large-seeded plants have much more rapid rates of decay than small, wind-dispersed seeds and that the rate of distance decay was significantly higher for fragmented forests than for contiguous forests. Subsequently, Soininen et al. [7] and Heino [47] suggested that a fruitful approach to evaluate the relative roles of species-sorting mechanisms, and dispersal limitation would be the comparison of the distance decay relationships produced by organisms with different dispersal abilities (considering data gathered at the same sites). Although Nekola and White [6] called our attention to the importance of dispersal ability in explaining the variation in the DDR more than a decade ago, this prediction has been tested only recently [11], [33]. These tests have been conducted in both aquatic and terrestrial systems and have focused on different taxonomic groups. Also, some interesting variations of the general prediction have been investigated, including the comparison of the distance decay rates between exotic and native species [48]–[51] and between parasitic communities of hosts with different vagilities [52].

Thus, we built on these works to formulate the main predictions of our study. Specifically, we predicted that groups of algae with higher dispersal ability should show lower rates of distance decay (or low scale-dependency of beta diversity according to Soininen et al. [7], when compared with groups with lower dispersal ability. As we found a higher distance decay rate for periphyton than for phytoplankton communities, our results support this prediction independently of the similarity index used (Simpson’s dominance index and Sørensen's similarity index for incidence data and Bray-Curtis index of similarity for abundance data). This pattern was also observed for the comparison between benthic and mobile algae within the periphyton communities. Brown and Swan [53] also found that only “low-dispersal communities” of macroinvertebrates inhabiting the main channels of a riverine network (Maryland, USA) exhibited significant DDR. Similarly, studies focusing on turnover patterns of exotic and native plant species in North America and Europe found that exotic species (supposedly with high dispersal ability) exhibited lower rates of similarity decay than do native species [48]–[50], [53]–[54]. In one extreme, Mazaris et al. [55] detected a significant DDR only for fish in a comparison with better dispersers’ communities (phytoplankton and zooplankton). However, the prediction of a lower rate of similarity decay for better dispersers did not hold in many cases [51]–[52], [56].

If we consider that body size is a reliable proxy for dispersal ability, and if we consider the analysis of the 401 DDR performed by Soininen et al. [7], then the generality of this prediction should be qualified. This would be because they did not detect a negative and significant relationship, as expected by the prediction, between body size and halving distance (a metric inversely related to the rate of distance decay). However, a possible caveat of this and all results described above, including ours, is that, instead of being directly measured, the trait “dispersal ability” is, in general, inferred from other traits, such as body size, growth form, habitat use, invasiveness, and, in the case of parasitic communities, host vagility. Thus, although we realize the methodological challenges of explicitly measuring dispersal ability for an entire community [57], especially for microorganisms, we suggest that a more reliable way to test the effect of dispersal ability on DDR would involve the direct measurement of dispersal.

Our study demonstrates that the comparison of distance decay relationships among diatom taxa with different dispersal abilities was a sound approach to evaluate the potential effects of dispersal rates on beta diversity patterns [9], [47]. Although dispersal ability is clearly high for microorganisms, the deconstructive approach we used suggested that a range of dispersal capabilities within this huge group could be found. The confirmation of our general prediction (higher rates of decay in community similarity for less-vagile algae groups) can be seen as an indirect validation of the suitability of the traits we used to create the groups. More importantly, these results indicate that treating all unicellular taxa as “microorganisms” may be a flawed approach to test whether microbes exhibit biogeographic patterns.

## Supporting Information

Figure S1 **Randomization test procedures.**
(PDF)

Figure S2 **Species richness of diatom families (** ***Bacillariophyceae*** **) registered in the Rio Negro hydrographical basin (Brazilian Amazon).**
(PDF)

Figure S3 **Simpson index applied to the incidence-based data.**
(PDF)

Table S1 **Number of quantified valves and life-form category of selected abundant species found in planktonic and periphytic communities in the Rio Negro hydrographical basin.**
(PDF)

Table S2 **Basic limnological data measured along the Negro river basin.**
(PDF)

Text S1 **Ecological definitions of life-forms and databases.**
(PDF)
