# Supplementary material for: Distance Decay of Similarity in Neotropical Diatom Communities
Source: PLoS One. 2012 Sep 13;7(9):e45071. doi: 10.1371/journal.pone.0045071 (PMC3441607; doi:10.1371/journal.pone.0045071)
Supplement: Figure S1 — Randomization test procedures. (PDF) [file pone.0045071.s001.pdf]

Supporting Information

Distance decay of similarity in Neotropical diatom communities

Carlos E. WETZEL, Denise de C. BICUDO, Luc ECTOR,

Eduardo A. LOBO, Janne SOININEN, Victor L. LANDEIRO and Luis M. BINI

Figure S1. Randomization test procedures.

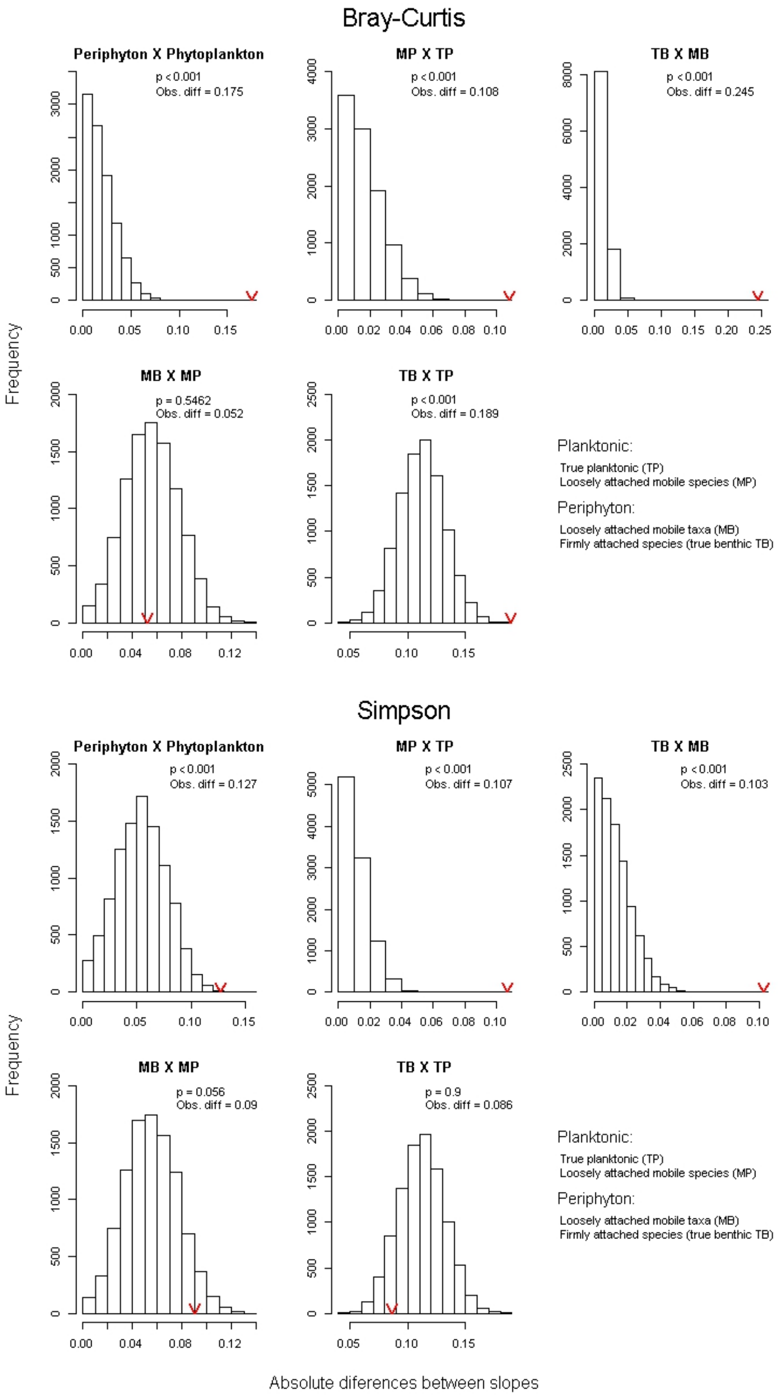

Results of the randomization test procedure (Monte Carlo) showing the null distributions of the differences between the slopes and the observed differences (red sign). P-values reported in the main text. Most of the differences were significant and in the direction predicted by us. In other words, the slope of the relationship between community similarity and geographic distance (i.e., distance decay rate) was significantly higher for the algal groups with low dispersal ability than for the algal groups with high dispersal ability (e.g., Total periphyton > Total phytoplankton, TB > MB and TB > TP).
