# Supplementary material for: Distance Decay of Similarity in Neotropical Diatom Communities
Source: PLoS One. 2012 Sep 13;7(9):e45071. doi: 10.1371/journal.pone.0045071 (PMC3441607; doi:10.1371/journal.pone.0045071)
Supplement: Figure S2 — Species richness of diatom families ( Bacillariophyceae ) registered in the Rio Negro hydrographical basin (Brazilian Amazon). (PDF) [file pone.0045071.s002.pdf]

Supporting Information

Distance decay of similarity in Neotropical diatom communities

Carlos E. WETZEL, Denise de C. BICUDO, Luc ECTOR,

Eduardo A. LOBO, Janne SOININEN, Victor L. LANDEIRO and Luis M. BINI

Figure S2. Species richness of diatom families[1] registered in the Rio Negro hydrographical basin (Brazilian Amazon).

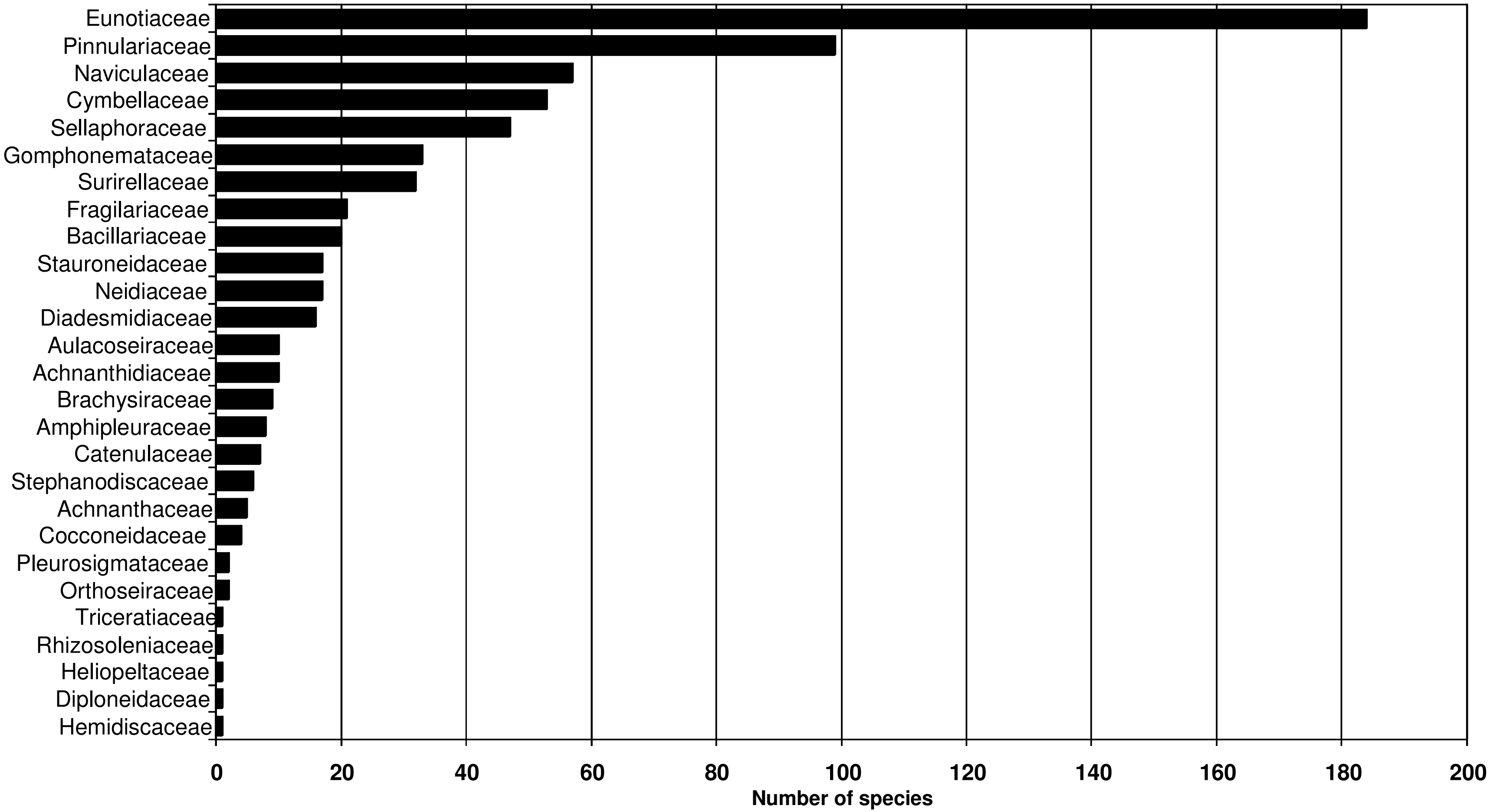

[1] Based on Round et al. (1990). *The Diatoms. Biology and morphology of the genera*. Cambridge University Press, 749 p.
