# Supplementary material for: Distance Decay of Similarity in Neotropical Diatom Communities
Source: PLoS One. 2012 Sep 13;7(9):e45071. doi: 10.1371/journal.pone.0045071 (PMC3441607; doi:10.1371/journal.pone.0045071)
Supplement: Figure S3 — Simpson index applied to the incidence-based data. (PDF) [file pone.0045071.s003.pdf]

Supporting Information

Distance decay of similarity in Neotropical diatom communities

Carlos E. WETZEL, Denise de C. BICUDO, Luc ECTOR,

Eduardo A. LOBO, Janne SOININEN, Victor L. LANDEIRO and Luis M. BINI

Figure S3. Simpson index applied to the incidence-based data.

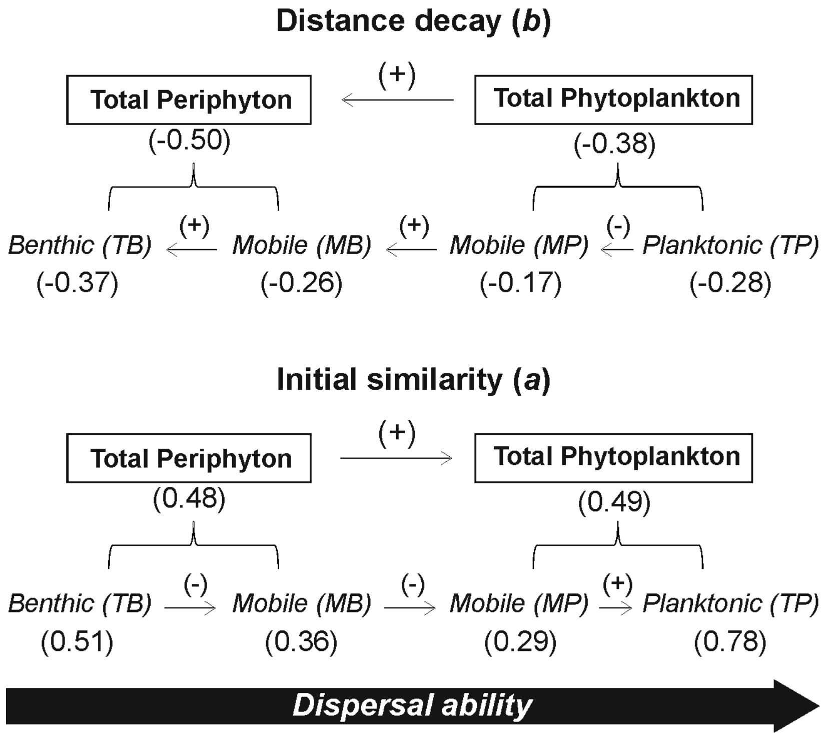

Distance decay (*b*) and intercepts (initial similarity "*a*") calculated for periphyton and phytoplankton communities using qualitative data. Positive (+) and negative (-) signs indicate whether our predictions were correct or incorrect, respectively.
