# Supplementary material for: Distance Decay of Similarity in Neotropical Diatom Communities
Source: PLoS One. 2012 Sep 13;7(9):e45071. doi: 10.1371/journal.pone.0045071 (PMC3441607; doi:10.1371/journal.pone.0045071)
Supplement: Table S1 — Number of quantified valves and life-form category of selected abundant species found in planktonic and periphytic communities in the Rio Negro hydrographical basin. (PDF) [file pone.0045071.s004.pdf]

Supporting Information

Distance decay of similarity in Neotropical diatom communities

Carlos E. WETZEL, Denise de C. BICUDO, Luc ECTOR,
Eduardo A. LOBO, Janne SOININEN, Victor L. LANDEIRO and Luis M. BINI

Table S1. Number of quantified valves and life-form category of selected abundant species found in planktonic and periphytic communities in the Rio Negro hydrographical basin. (TP = true planktonic; MP/MB = loosely attached mobile species; TB = firmly attached species, true benthic).

Phytoplankton	Number of counted valves	Life form
Aulacoseira granulata (Ehrenberg) Simonsen	16.236	TP
Eunotia tukanorum C.E. Wetzel & D. Bicudo	11.943	TP
Eunotia parasiolii Metzeltin & Lange-Bertalot	5.049	TB
Eunotia loboi C.E. Wetzel & Ector	3.542	TP
Eunotia gomesii C.E. Wetzel & Ector	1.930	TP
Fragilaria javanica Hustedt	1.873	TP, MB
Aulacoseira herzogii (Lemmermann) Simonsen	1.741	TP
Eunotia waimiriorum C.E. Wetzel	1.694	TP

Periphyton	Number of counted valves	Life form
Eunotia parasiolii Metzeltin & Lange-Bertalot	12.570	TB
Eunotia souzae Metzeltin & Lange-Bertalot	3.404	TB
Eunotia botuliformis Wild, Nörpel & Lange-Bertalot	3.008	TB
Eunotia trinacria Krasske	2.799	TB
Eunotia mucophila (Lange-Bertalot & Nörpel-Schempp) Lange-Bertalot	2.662	TB
Fragilaria javanica Hustedt	2.532	TP, MB
Eunotia sulcatoides Metzeltin & Lange-Bertalot	1.508	TB
Nupela rumrichorum Lange-Bertalot	1.466	MB
