# Supplementary material for: Distance Decay of Similarity in Neotropical Diatom Communities
Source: PLoS One. 2012 Sep 13;7(9):e45071. doi: 10.1371/journal.pone.0045071 (PMC3441607; doi:10.1371/journal.pone.0045071)
Supplement: Table S2 — Basic limnological data measured along the Negro river basin. (PDF) [file pone.0045071.s005.pdf]

Supporting Information

Distance decay of similarity in Neotropical diatom communities

Carlos E. WETZEL, Denise de C. BICUDO, Luc ECTOR,
Eduardo A. LOBO, Janne SOININEN, Victor L. LANDEIRO and Luis M. BINI

Table S2. Basic limnological data measured along the Negro river basin.

Basic limnological data measured along the Negro river basin: pH (n = 41), temperature (˚C) (n = 41), dissolved oxygen (DO = mg L^{-1}) (n = 41), and conductivity (COND = µS cm^{-1}) (n = 29).

	pH	Temp.	DO	COND
**Mean**	4.5	26.8	4.3	17.98
**S.D.**	0.5	1.5	1.6	9.75
**Min.**	3.8	25.0	2.1	8.00
**Max.**	6.6	30.0	8.0	41.80
