# Supplementary material for: Distance Decay of Similarity in Neotropical Diatom Communities
Source: PLoS One. 2012 Sep 13;7(9):e45071. doi: 10.1371/journal.pone.0045071 (PMC3441607; doi:10.1371/journal.pone.0045071)
Supplement: Text S1 — Ecological definitions of life-forms and databases. (PDF) [file pone.0045071.s006.pdf]

Supporting Information

Distance decay of similarity in Neotropical diatom communities

Carlos E. WETZEL, Denise de C. BICUDO, Luc ECTOR,
Eduardo A. LOBO, Janne SOININEN, Victor L. LANDEIRO and Luis M. BINI

Text S1. Ecological definitions of life-forms and databases.

***Benthic spp. (TB)*:** The data table consists of diatoms tightly adnated to the substrate ('monoraphid' taxa such as *Achnanthes* Bory de Saint-Vincent, *Cocconeis* Ehrenberg and *Planothidium* Round et Bukhtiyarova) and pedunculated or stalked diatoms that include 'brachyraphid', 'araphid' and 'biraphid' diatom taxa (*e.g., Eunotia* Ehrenberg*, Fragilaria* Lyngbye*, Fragilariforma* D.M. Williams et Round and *Gomphonema* Ehrenberg spp.) that are attached by means of generally short stalks, including prostrate (adhering to the substrate with the entire valve surface), adnate (apically attached but parallel to the substrate) and, erect (apically attached but perpendicular to the substrate).

Adnated and small mobile diatoms are part of the same continuum of small prostrate diatoms that have high adherence and lower dispersion rates (low-risk strategy) for higher motility but higher resuspension/dispersal rates. Species that meet these criteria in the Amazon are members of the genus *Nupela* Vyverman et Compère and *Chamaepinnularia* Lange-Bertalot et Krammer. Most of the Cymbellaceae family was included in this group. Benthic species found on phytoplankton assemblages were removed from this group because their occurrences should be occasional.

Mobile spp. (MP and MB): Mobile diatoms are included on both the phytoplanktonic and periphytic matrices because they were found either on benthic communities or drifting on the water column. They include loosely attached diatoms with no obvious method of attachment. This group mainly consists of the large mobile species and slow-moving species belonging to the genera *Pinnularia* Ehrenberg, *Stauroneis* Ehrenberg*, Surirella* Turpin, *Gyrosigma* Hassall, *Navicula* Bory de Saint-Vincent and *Nitzschia* Hassall. The strong flood pulse of the Negro River and its tributaries could resuspend sedimented diatoms and littoral periphyton, increasing not only the number of species, but also the number of functional groups represented in the planktonic community.

Planktonic spp. (TP): This group is mainly composed of truly free-floating planktonic species (*e.g.*, *Aulacoseira* Thwaites, *Tabellaria* Ehrenberg *ex* Kützing and *Urosolenia* Round et R.M. Crawford). Tychoplanktonic species, which are easily resuspended from substrates to the water column (*i.e.*, centric diatoms belonging to Stephanodiscaceae, such as *Cyclotella* (Kützing) Brébisson, *Discostella* Houk et Klee and *Stephanodiscus* Ehrenberg) are included in this category. Species belonging to the *Eunotia asterionelloides* Hustedt species-complex are included on this group because they were among the most representative of this hydrographical basin (Wetzel *et al.*, 2010).

References Text S1:

Database was explored using usually the genus level instead of species level. The information was gathered and builds on several previous publications that are summarized on the following articles:

Hoagland KD, Roemer SC, Rosowski JR (1982) Colonization and community structure of two periphyton assemblages, with emphasis on the diatoms (Bacillariophyceae). Am J Bot 69: 188-213.

Passy SI (2007) Diatom ecological guilds display distinct and predictable behavior along nutrient and disturbance gradients in running waters. Aquat Bot 86: 171-178.

Ribeiro LLCS (2010) Intertidal benthic diatoms of the Tagus estuary: Taxonomic composition and spatial-temporal variation. PhD Thesis, Universidade de Lisboa, Portugal.

Rimet F, Bouchez A (2011) Use of diatom life-forms and ecological guilds to assess pesticide contamination in rivers: Lotic mesocosm approaches. Ecol Ind 11: 489-499.

Rimet F, Bouchez A (2012) Life-forms, cell-sizes and ecological guilds of diatoms in European rivers. Knowledge and Management of Aquatic Ecosystems 406: DOI: 10.1051/kmae/2012018

Wetzel CE, Ector L, Hoffmann L, Bicudo DC, (2010) Colonial planktonic *Eunotia* (Bacillariophyceae) from Brazilian Amazon: taxonomy and biogeographical considerations on the *E. asterionelloides* species complex. Nova Hedwigia 91: 49-86.
